# Relationship between caudal fin closing motion and acceleration capability of *Rudarius ercodes* balistiform locomotion

**DOI:** 10.1038/s41598-025-00315-9

**Published:** 2025-05-08

**Authors:** Takehiro Fuji, Hiroaki Sumikawa, Naoya Hirata, Yoshihiro Kimori, Masafumi Kato, Takashi Fukue

**Affiliations:** 1 Department of Mechanical Engineering, Graduate School of Engineering, Kanazawa Institute of Technology, 3-1 Yatsukaho, Hakusan, Ishikawa 924-0838 Japan; 2 Mechanical and Aerospace Engineering, Division of Science and Engineering, Iwate University, 4-3-5 Ueda, Morioka, Iwate 020-8551 Japan; 3 Notojima Aquarium, 15-40 Notojimamagarimachi, Nanao, Ishikawa 926-0216 Japan; 4 Department of Mechanical Engineering, College of Engineering, Kanazawa Institute of Technology, 3-1 Yatsukaho, Hakusan, Ishikawa 924-0838 Japan

**Keywords:** Locomotion, Balistiform, CFD, 3D self-propulsion analysis, *Rudarius ercodes*, Fluid dynamics, Behavioural ecology

## Abstract

Fish leverage the interaction of multiple fins to improve their swimming ability. Acceleration performance, in particular, is a key aspect of locomotion that directly affects survival through predator evasion and prey capture. Most of these studies have focused on body and caudal fin (BCF) swimmers, which are specialized for high-speed locomotion. In contrast, relatively few studies have examined median and paired fin (MPF) swimmers, and fins’ functional roles under different locomotor modes remain insufficiently understood. Therefore, MPF swimmers may employ acceleration strategies distinct from those of BCF swimmers. Understanding the effects of fin interaction on acceleration performance in MPF swimming can contribute to a unified understanding of how such interactions influence swimming performance across fishes with different locomotor modes. A type of MPF swimmer is the balistiform, which uses its dorsal and anal fins as the main propulsion organs. We observed the closing of the caudal fins in *Rudarius ercodes* in balistiform locomotion during acceleration. We hypothesized that *R. ercodes* increased their average acceleration (the time taken to reach the experimentally observed velocity) by closing their caudal fins. We performed water tank observations to capture swimming behavior and three-dimensional computational fluid dynamics (3D-CFD) analysis to clarify the impact of caudal fin opening and closing on acceleration capability in balistiform locomotion. For example, parameters such as swimming speed and caudal fin spreading angle were measured in the swimming observations. In contrast, the fluid dynamic analysis computed the vortex structures, propulsive efficiency (the ratio of input energy contributing to thrust), and the cost of transport (the energy required to travel a unit distance). Our analysis showed that closing the caudal fin increased the average acceleration by 30%, increased the cost of transport, and decreased the dimensionless Froude efficiency. We also clarified the role of the caudal fin in MPF locomotion straight-line swimming. Our findings will help us better understand how fin interactions affect fish’s swimming ability.

**Supplementary Information:**

The online version contains supplementary material available at 10.1038/s41598-025-00315-9.

## Introduction

Fish morphology and swimming behavior have been optimized by natural selection to promote adaptation to their ecosystem and habitat. Fish swimming ability plays an important role in migration, habitat selection, predator-prey interactions, and reproduction^1^. For example, it has been shown that superior acceleration capability is necessary for evading predatory fish^2^. It is crucial to explore how morphology and locomotion methods affect swimming ability to comprehend fish ecology^3,4^. Understanding fish swimming ability is essential not only for ecological studies but also for engineering research. Because fish’s swimming ability is superior to that of existing underwater robots, the high-performance swimming ability of fish has captivated researchers in various fields, including biologists, ecologists, and robotics engineers^5–9^.

Fish swimming methods are broadly classified into two categories by Lindsey^10^: BCF locomotion using the body and caudal fins and MPF locomotion using the pectoral, dorsal, and anal fins. Studies on the mechanism of fish swimming have shown that the inverse Karman vortex contributes significantly to the generation of thrust in BCF and MPF locomotion^11,12^.

There are reports that multiple fins interactions affect acceleration performance^13^, maneuverability^14,15^, and propulsion efficiency^16–18^. In BCF fish, it has been reported that the presence or opening of the anal and dorsal fins enhances the vortex generated by the caudal fin^16,19,20^. As a result, it has been found that opening the anal and dorsal fins increases energy consumption but also improves swimming efficiency and acceleration. On the other hand, in MPF fish, there have also been reports of interactions between the anal, the dorsal and the caudal fins. In MPF swimming, it has been found that the stability of swimming can be improved by adjusting the phase difference between the anal, the dorsal and the caudal fins^11,15^.

As a type of MPF locomotion, the balistiform locomotion in *Rudarius ercodes* involves swimming with dorsal and anal fins while keeping the body and caudal fins steady^21^. We observed the opening of the caudal fin during hovering and turning movements and the closing of the caudal fin during acceleration in *R. ercodes* (supplementary video 1). Studies have indicated that fin opening and closing influence fish swimming ability. Sun et al.^19^ have indicated that raising the dorsal and anal fins in BCF locomotion fish strengthens the backward vortices, resulting in an 85% improvement in acceleration performance. The erection of median fins enhances turning maneuvers when the initial swimming velocity increases^22^. Regarding MPF locomotion, it has also been shown that yellow boxfish use pectoral, dorsal, and caudal fins to stabilize their hydrodynamically unstable body trajectory and control turning maneuvers^23^. Webb^24^ reported that MPF locomotion fish controlled their swimming stability by caudal fin opening or closing. However, the influence of caudal fin opening and closing on the acceleration capability in MPF locomotion is unclear. We hypothesized that closing the caudal fin would increase acceleration capability in straight-line swimming because *R. ercodes* closed their caudal fins during acceleration. The acceleration capability of fish markedly impacts survival^25^. Therefore, it is essential to investigate how caudal fin opening and closing influence acceleration in MPF locomotion.

This study aims to clarify the relationship between caudal fin closing motion and acceleration capability in straight-line swimming balistiforms. We evaluate the hydrodynamic performance of *R. ercodes* through a combination of water tank observation and self-propulsion analysis of water flow around the *R. ercodes* 3D model in which the dorsal and anal fins move. We hypothesized that *R. ercodes* improve their average acceleration by closing their caudal fins. To verify our hypothesis, we examined two questions: (1) How did the opening and closing of the caudal fin affect the acceleration capability? and (2) what the mechanism is.

## Methods

### Animal husbandry and ethics statement

**Fig. 1:**
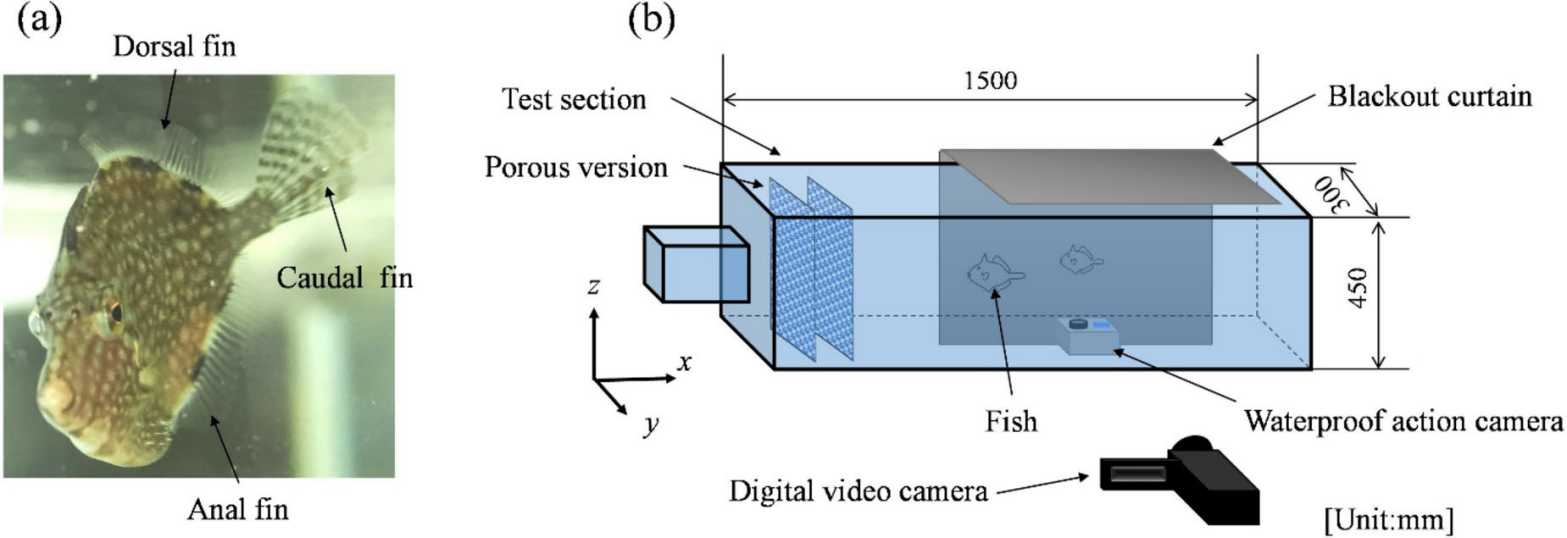
(**a**) *Rudarius ercodes*. (**b**) Schematic diagram of the experimental apparatus.

Experiments were conducted at the Yatsukaho Research Campus of Kanazawa Institute of Technology, in compliance with the Animal Welfare Law and related regulations, and after verifying Kanazawa Institute of Technology’s Regulations for the Management of Animal Experiments. The institute’s animal experiment regulations state that the experimental animals are reptiles, birds, and mammals. Since the present study involved only fish, we consulted with the Research Promotion Division of Kanazawa Institute of Technology before conducting the experiments. The division confirmed that the experimental protocol did not fall under the category of animal experiments requiring approval by the Animal Experiment Committee, following the institute’s Regulations for the Management of Animal Experiments—which apply only to reptiles, birds, and mammals—and could therefore be conducted without ethical concerns. Moreover, this study was conducted in compliance with the guidelines for the use of fish in research published by the Ichthyological Society of Japan in 2003, and particular attention was paid to breeding management and pain reduction when fish were used in water tank observation. This manuscript was written following ARRIVE guidelines. We collected *R. ercodes* in Nanao Bay, Noto, Japan (Fig. 1 (a)). The collected *R. ercodes* were kept in experimental tanks for over one year to acclimate to the environment. The experimental tank was also designed for rearing, and observation and rearing were conducted in the same tank. The water temperature was approximately 24 ^o^C, and the specific gravity of artificial seawater ranged from 1.021 to 1.024. Feeding was done at least once every two days, twice daily in the morning and the afternoon.

### Water tank observation

*R. ercodes* movement was observed and recorded on video. Figure 1 (b) shows a schematic diagram of the water tank and the photography equipment used in the observation. The working fluid was artificial seawater at 24 °C. *R. ercodes* were allowed to swim in a tank (1500 mm wide, 300 mm deep, 450 mm high) filled with artificial seawater to evaluate locomotion behavior. The experiments were specifically designed to determine under what form of straight-line swimming *R. ercodes* open and close their caudal fins and to measure the number of dorsal and anal fin oscillations caused by the opening and closing of the caudal fins. A digital camera was used to capture 60 fps video from the front. In addition, a 60 fps video was shot underwater with an action camera. Two *R. ercodes* of different sizes (total body length (*BL*): 45: mm and 57 mm) were targeted for observation. Each *R. ercodes* was photographed ten times for a total of 20 shots.

### Calculation of swimming velocity

In this research field, DeepLabCut™ is often used to analyze captured video^26–28^. DeepLabCut is a pose estimation algorithm for detecting animal body parts created by Mathis et al.^29^. To calculate *R. ercodes* swimming velocity, we used DeepLabCut to, track and quantify the body part to be evaluated by deep learning training data. The three videos taken in the experiment were used as training data. Next, labels were manually set on the mouthparts to generate training data. Thereafter, the labeled training data were trained 5,000 times for the instrumentation. These training data and networks were used to calculate other videos. The relationship between the time required to reach the experimentally measured velocity and the calculated velocity is evaluated as the average acceleration *α* [*BL*/s^2^]:

1 $$\:\alpha\:=\frac{{u}_{\text{e}}}{{t}_{0}}$$,

where *u*_e_ [*BL*/s] is the swimming velocity of the locomotion measured in the swimming experiment, and *t*_0_ [s] is the time required to reach *u*_e_ in the CFD analysis.

### CFD analysis

We used OpenFOAM^®^ to perform a self-propulsion analysis of fluid dynamics^30^. OpenFOAM is an open-source program that is free of charge and easy to modify, making it accessible to many people who want to get involved in the fluid dynamics analysis of fish. It also has a proven track record in fluid dynamics analysis of fish, including our previous researches^31–34^. Furthermore, in this study, we performed a self-propulsion analysis to calculate the transient response of acceleration.

**Fig. 2:**
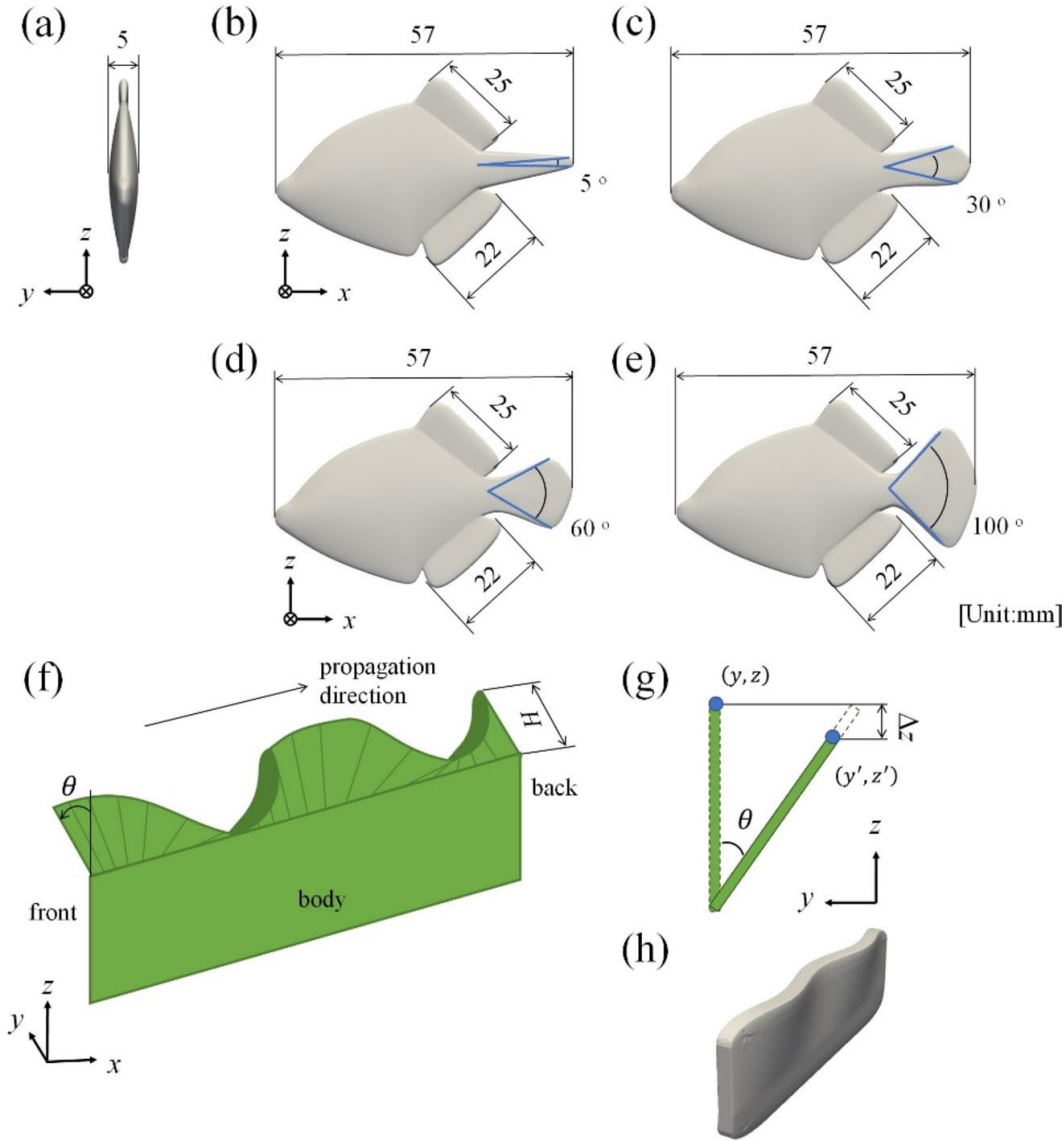
Schematic of CFD model. (**a**) and (**b**) show the model of the caudal fin closed. (**c**) and (**d**) show the model of the caudal fin open. (**e**), (**f**), (**g**), and (**h**) are given motion 3D models.

**Fig. 3:**
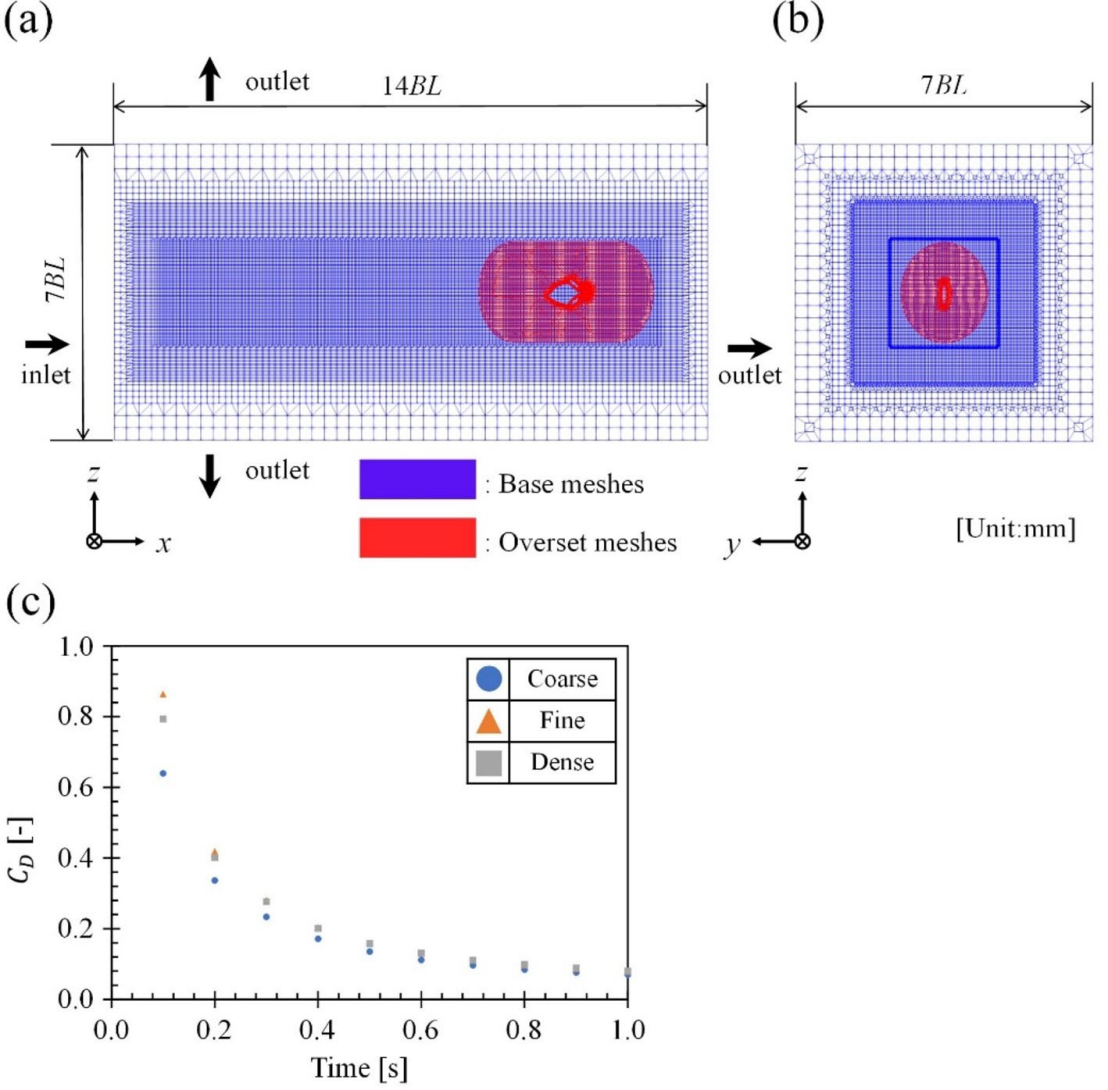
Meshes for 3D-CFD analysis and differences in force between meshes. (**a**) shows meshes on the coronal plane of the whole fluid region. (**b**) shows the frontal cross-section of the fluid region in (**a**). The red region is the overset mesh region. (**c**) shows *R. ercodes* instantaneous drag coefficient vs. using coarse, fine, and dense meshes.

Figure 2 (a), (b), (c), (d) and (e) show the 3D models of *R. ercodes* created for CFD analysis. Figure 2(a) shows the *R. ercodes* from the front, and the width is the same regardless of the open angle of the caudal fin. Figure 2 (b) reproduces an *R. ercodes* with its caudal fin closed, and Fig. 2 (e) reproduces an *R. ercodes* with its caudal fin full-open. Here, the caudal fin was defined as full-open when it was more than 90 ^o^ open, and a model was created under the frequently observed condition that the caudal fin was 100 ^o^ open. On the other hand, a less than 20 ^o^ open caudal fin was defined as closed, and a model was created under the frequently observed 5 ^o^ closed condition. In addition, we also created 30° and 60° open caudal fin models to investigate the effect of the angle of the caudal fin opening. However, please note that these two opening angles were not observed in the observation and were created to verify the effect of the opening angle. The 3D models of *R. ercodes* (total length: 57 mm, total width: 7 mm, total height: 40 mm, symmetrical structure, dorsal and anal fins oscillate separately for locomotion) were developed by Blender 2.93.6^35^. , an open-source 3D creation tool. A frequency (*f*) higher than the average frequency of 14 Hz was considered fast swimming, and *f* lower than 14 Hz was considered slow swimming. The CFD analysis selected an average *f* of 29 Hz for fast swimming and an average *f* of 10 Hz for slow-speed swimming. Figure 3 (a) and (b) show the schematic of the mesh construction of the proposed CFD analysis model. As a solver of the CFD analysis, OpenFOAM^®^ v.1806^30^, an open-source FVM-CFD solver, was used. The developed 3D model of *R. ercodes* was installed in the CFD analysis model. The overset grid function represented the movement of *R. ercodes* dorsal and anal fins. The overset framework is a versatile way of using overset meshes, which can be applied to static and dynamic scenarios. It involves creating mappings between different mesh regions that may not be directly connected, resulting in a combined domain. This approach allows for complex mesh movements and interactions without the drawbacks of deforming meshes and is handy for treating single- and multiphase flows^36^. The base structural meshes constructed in the calculation are shown in blue in Fig. 3 (a) and (b). The overlapping grid around *R. ercodes* is shown in red in Fig. 3 (a) and (b) were constructed by snappyHexMesh, an unstructured grid generation function. The governing equations are the continuity equation and the three-dimensional incompressible Navier–Stokes equation:

2 $$\:\nabla\:\cdot\:u=0$$

3 $$\:\frac{\partial\:u}{\partial\:t}+\nabla\:\cdot\:\left(uu\right)=-\nabla\:p+\nabla\:\cdot\:\left(\nu\:\nabla\:u\right)+\nabla\:\cdot\:\left[\nu\:\left\{{\left(\nabla\:u\right)}^{T}-\frac{1}{3}\nabla\:\cdot\:uI\right\}\right],$$

where *u* [m/s] is the velocity vector, *t* [s] is the time, *p* [m^2^/s^2^] is the static pressure divided by the reference density, *ν* [m^2^/s] is the kinematic viscosity, and *I* is the unit tensor.

Figure 2 (g) shows an image of fin locomotion^37^. Equation (5) denotes the amount of fin movement in the *y*-axis direction. Coefficient *β* in Eq. (6) is applied to the amount of fin movement in the *y*-axis direction in the first cycle when the mesh begins to move to prevent divergence of the calculation due to a large amount of movement. As shown in Fig. 2 (h), the length of the fin is kept constant by applying Eq. (7), which considers the travel elongation in the *z*-axis direction of Δ*z*. Equation (8) denotes the amount of fin movement in the *z*-axis direction. The created motion is shown in Fig. 2 (i) and supplementary video 2:

4 $$\:\theta\:=\left\{\begin{array}{c}{\beta\:\cdot\:\theta\:}_{\text{m}\text{a}\text{x}}sin\left[2\pi\:\left(\frac{x}{\lambda\:}+ft\right)\right]\:\:\:\left(t\:\le\:T\right)\\\:{\theta\:}_{\text{m}\text{a}\text{x}}sin\left[2\pi\:\left(\frac{x}{\lambda\:}+ft\right)\right]\:\:\:\:\:\:\:\:\:\left(T\:<t\right)\end{array}\right.$$

5 $$\:{y}^{{\prime\:}}=z\text{sin}\theta\:+y$$

6 $$\:\beta\:=\frac{{\left(t-T\right)}^{4}+{T}^{4}}{{T}^{4}}$$

7 $$\:\varDelta\:z=2\:z\:\text{cos}\left(\frac{\pi\:-\theta\:}{2}\right)\text{sin}\left(\frac{\theta\:}{2}\right)$$

8 $$\:{z}^{{\prime\:}}=z-\varDelta\:z,$$

where *x* [m] is the *x*-coordinate of any point, *y* [m] is the *y*-coordinate of any point before movement, *y*’ [m] is the *y*-coordinate of any point after movement, *z* [m] is the *z*-coordinate of any point before movement, *z*’ [m] is *z*-coordinate of any point after movement, *θ* [^o^] is the fin angular excursion, *θ*_max_ [^o^] is the peak amplitude, *λ* [m] is the wavelength, *f* [Hz] is the undulation frequency, *H* [m] is *R. ercodes* fin height, *T* [s] is the cycle, and *t* [s] is the time. Although the shape of the 3D model (opening and closing of the caudal fin) was changed, the same motion was given to the dorsal and anal fins.

Next, a 3D self-propulsion analysis was performed to calculate the amount of movement based on the forces acting on the 3D model surface, considering the reaction and inertia forces from the fluid in each calculation step, which were calculated by numerical analysis. Equation (11), which discretizes the equations of motion shown in Eqs. (9) and (10) using the second-order backward difference method, was used to calculate the amount of movement, as in the previous study^38^:

9 $$\:m\frac{d{u}_{\text{a}}}{dt}=F$$

10 $$\:F=\left[{\int\:}_{s}^{\:}\left(-P{n}_{1}+{\tau\:}_{\text{i}\text{j}}{n}_{\text{j}}\right)ds\right]$$

11 $$\:\frac{3{{u}_{\text{a}}}^{n+1}-4{{u}_{\text{a}}}^{n}+{{u}_{\text{a}}}^{n-1}}{2\varDelta\:t}=\frac{{F}^{\text{n}+1}}{m},$$

where *m* [kg] is the mass (0.0026 kg), *u*_a_ [m/s] is the swimming velocity of the 3D model in the *x*-axis direction, *F* [N] is the *x*-axis force acting on the 3D model, *s* [m^2^] is the total surface area, *ds* [m^2^] is the small surface area, *P* is the pressure vector acting on the small area, *n*_j_ is the unit vector of the *j*th element, τ_ij_ is the stress tensor acting on the small area. Since *R. ercodes* exhibited minimal lateral motion, a self-propelled simulation was performed with degrees of freedom released only in the x-direction to reduce computational cost. The stresses were derived using Newton’s viscosity law. The validity of the self-propulsion method was verified by comparing it with the studies of Kern and Koumoutsakos^39^ and Huangs et al.^40^. The same undulating motion as in the previous study was applied to the same simple fish-shaped model and compared with the swimming velocities reported by Kern and Koumoutsakos^39^ and Huangs et al.^40^. The error between the swimming velocity obtained by this method and those in the two previous studies was 5%. Detailed information on the verification of this analysis method is provided in Supplementary Information.

The Reynolds number was defined in a previous study^41^:

12 $$\:{R}_{\text{e}}=\frac{{u}_{\text{e}}{L}_{D}}{\nu\:}$$,

where *L*_D_ [m] is the length of the 3D models and, *ν* [m^2^/s] is the kinematic viscosity of artificial seawater at 24 °C (9.5818 × 10^–7^ m^2^/s). The average Reynolds number in this study is 4637. Considering this, we used the laminar model in OpenFOAM. This model is a templated abstract base class for laminar transport models in OpenFOAM^42^. The laminar model calculates the small fish locomotion flow^43–45^. The instantaneous drag coefficient vs. time plot with mesh size as a parameter is shown in Fig. 3 (c). Analyses were performed on three meshes: coarse, fine, and dense. The coarse mesh had 1.5 × 10^6^ elements; the fine mesh, 2.9 × 10^6^ elements; and the dense mesh, 4.4 × 10^6^ elements. Analyses were performed on *R. ercodes* with a fin *f* of 29 Hz and a closed caudal fin. As shown in Fig. 3 (c), the drag coefficient is almost the same when the mesh is fine and when the mesh is dense. The mean drag coefficient error of fine and dense meshes is 6.0%. The fine mesh was used in all simulation cases in consideration of accuracy. We used the same meshes for all simulation cases. This research used the FUJITSU Supercomputer PRIMEHPC FX1000 and FUJITSU Server PRIMERGY GX2570 (Wisteria/BDEC-01) at the Information Technology Center, The University of Tokyo.

### Analytical conditions

As the boundary condition, non-slip wall boundaries were used for *R. ercodes* surface, and free flow-out boundaries were used for the water tank. The parameters of the 3D model were based on *R. ercodes* with closed caudal fins obtained from the water tank observation. In this case, the traveling waves in the dorsal and caudal fins are in the same positive *x*-axis direction. Analyses were conducted with two conditions: the inflow velocity of 0.0 m/s and two types of caudal fin (Fig. 2 (a) to (f)). With energetics being an essential factor during the routine activities of fish^46^. To study fluid dynamics energy expenditure, we evaluated two indexes, the dimensionless Froude efficiency *η* [-] and the cost of transport *Ω* [J/kg m]^46^:

13 $$\:\eta\:=\frac{{T}_{f}{u}_{\text{a}}}{{P}_{e}}$$

14 $$\:\varOmega\:=\frac{{P}_{e}}{{u}_{\text{a}}m}$$,

15 $$\:{P}_{e}={\int\:}_{0}^{{T}_{20}}\left[{\varSigma\:}_{s}\{{f}_{x}\frac{dx}{dt}+{f}_{y}\frac{dy}{dt}+{f}_{z}\frac{dz}{dt}\}\right]dt$$

where $\:{T}_{f}$ [N] is the *x*-axis thrust acting on the 3D model, and $\:{P}_{e}$ [W] is energy consumption. $\:{f}_{x}$, $\:{f}_{y}$, and $\:\:{f}_{z}$ are the forces in each axis direction applied to a small area of the *R. ercodes* surface, $\:\frac{dx}{dt}$, $\:\frac{dy}{dt}$, and $\:\frac{dz}{dt}$ are the velocities of movement in each axis direction of a small area of the *R. ercodes* surface, $\:{T}_{20}$ is the total duration spanning 20 cycles.

Drag coefficient is expressed using *C*_d_ [-]:

16 $$\:{C}_{\text{d}}=\frac{D}{\frac{1}{2}\rho\:{U}_{\text{a}}s}$$

where *D* [N] is the drag and, *ρ* is the density of artificial seawater at 24 °C (1023.6881 kg/m^3^). The following is an equation that shows the relationship between the *x*-axis force, the *x*-axis thrust and drag:

17 $$\:F={T}_{f}-D\:$$

## Results

In the following results, we first describe the observed differences in swimming speed, followed by analyses of swimming cost and propulsive efficiency. We then investigate the underlying vortex structures and finally evaluate the hydrodynamic forces (drag, thrust, and net thrust).

### Observation of *R*. *ercodes* swimming behavior

**Fig. 4:**
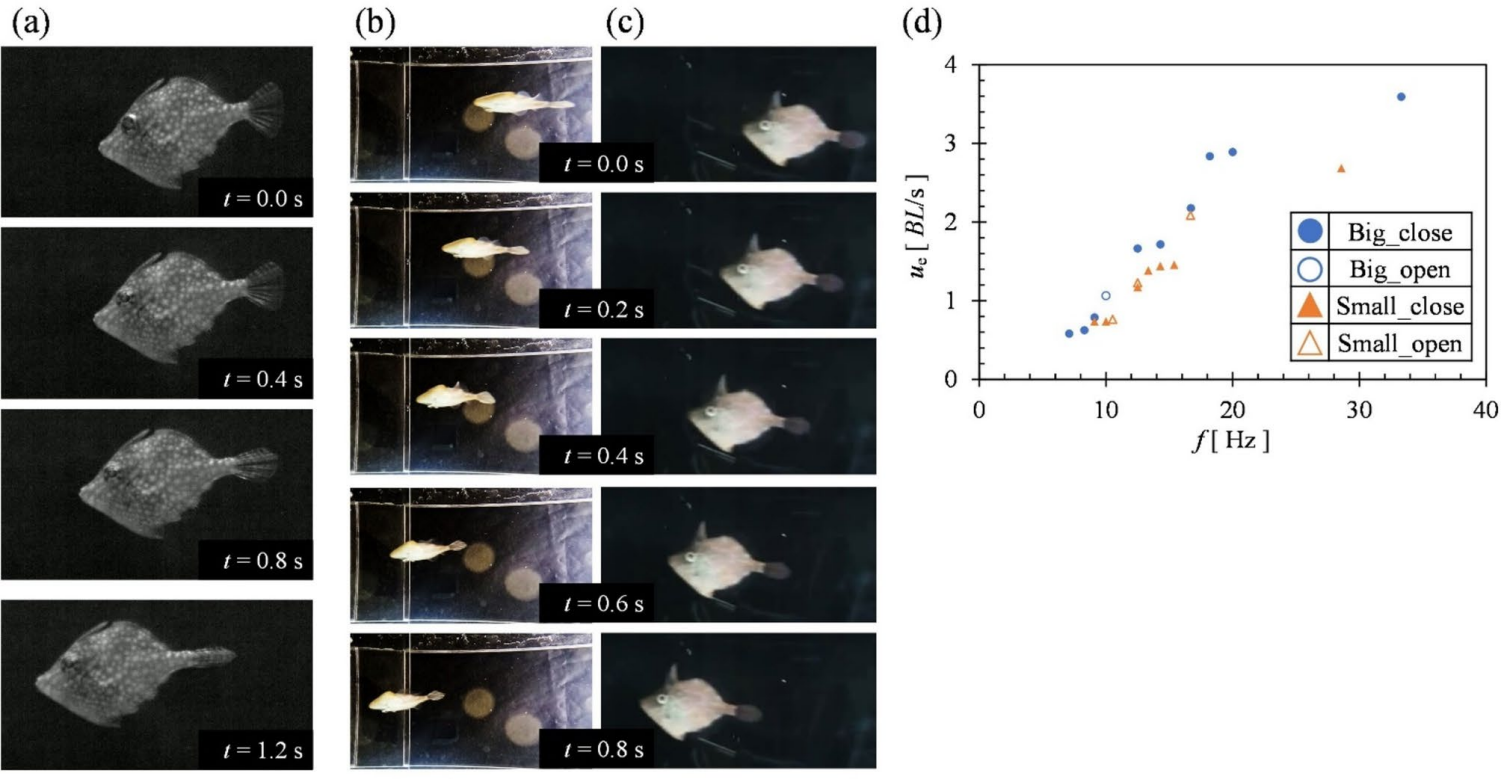
*R. ercodes* swimming behavior when making a straight line. (**a**) shows *R. ercodes* closing their caudal fins for acceleration. (**b**) shows underwater photographs of fish underside. (**c**) shows photographs taken from the side view. (**d**) is a plot of *f* vs. *u*_e_. Circles indicate *R. ercodes* with a total length of 0.057 m and triangles indicate *R. ercodes* with a total length of 0.045 m. Filled symbols represent the caudal fin closed, and unfilled ones represent the caudal fin open.

As shown in Fig. 4 (a), *R. ercodes* closed its caudal fin for acceleration. Two swimming behavioral patterns were captured on video: one with the caudal fin open and the other with the caudal fin closed. The value of *f* changed as the swimming velocity (referred to as *u*_e_) changed, while the amplitude and the wavelength remained unchanged, consistent with the results of Zhu et al.^47^. Figure 4 (b) and (c) show how *R. ercodes* performed linear swimming. As shown in Fig. 4 (c), only the dorsal and anal fins oscillated during swimming, tilting in the same direction. This is also seen in MPF swimmers with similar locomotion behavior, such as *mola mola*^48^. Figure 4 (d) shows the relationship between *f* and *u*_e_. The two *R. ercodes* were often observed to oscillate their fins at *f* at 10 to 20 Hz, with sudden increases in *f* to around 30 Hz (Fig. 4 (d)). Furthermore, a linear positive correlation between oscillation *f* and *u*_e_ was strongly noted. This result is similar to *f* and *u* trends of MPF fish studied by Kato^49^.

### Swimming velocity and fluid dynamic energetic expenditure

**Fig. 5:**
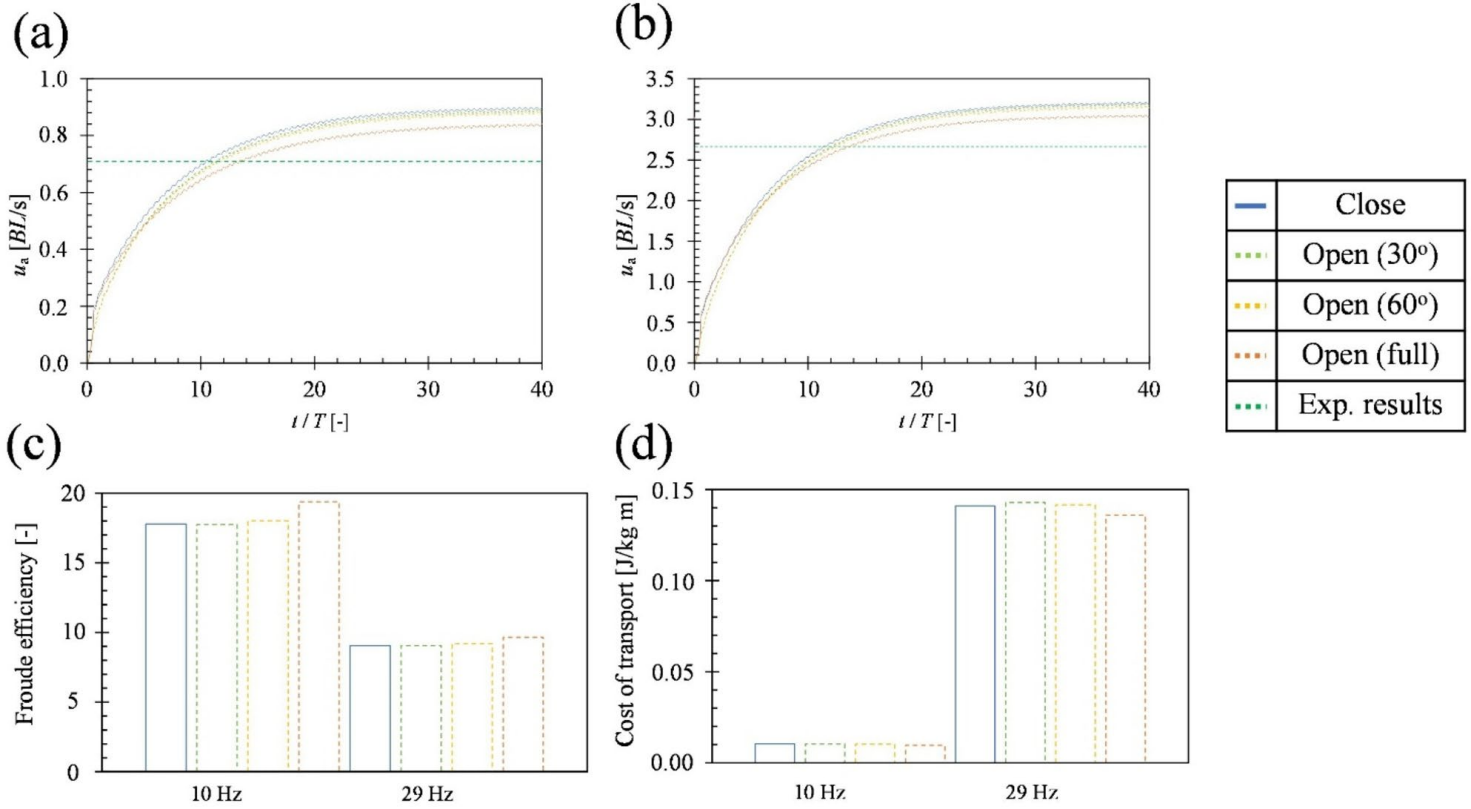
(**a**) and (**b**) show comparisons of *u*_a_ between open and closed caudal fins. (**c**) shows comparisons of Froude efficiency between open and closed caudal fins. (**d**) shows comparisons of cost of transport between open and closed caudal fins. In (**a**), *f* of the dorsal and anal fins is 10 Hz. In (**b**), *f* of the dorsal and anal fins is 29 Hz.

Figure 5 (a) shows the variation of *u*_a_ with time when *f* was 10 Hz. The average acceleration was 0.64 *BL*/s^2^ with the closed caudal fin, 0.59*BL*/s^2^ with the 30^o^ open caudal fin, 0.58*BL*/s^2^ with the 60^o^ open caudal fin, and 0.49 *BL*/s^2^ with the full-open caudal fin (Fig. 5(a)). Acceleration performance increased as the opening angle of the caudal fin decreased. The *u*_a_ difference between closed and full-open caudal fin is 30%. To reach the experimentally measured *u*_e_ (0.73 *BL*/s) (Fig. 5(a)), 11.4 cycles were required with the caudal fin closed and 14.9 cycles were required with the full-open caudal fin(Fig. 5(a)). The difference between the full-open and closed caudal fins was 3.5 cycles, which was 0.35 s, and the difference in swimming distance during this period was 0.2 *BL*. Furthermore, a difference of 0.06 *BL*/s was observed between the termination velocity *u*_a_ of the closed and full-open caudal fin (Fig. 5(a)). Figure 5 (b) shows the variation of *u*_a_ with time velocity when *f* was 29 Hz. The average acceleration was 6.73 *BL*/s^2^ with the closed caudal fin, 6.43*BL*/s^2^ with the 30^o^ open caudal fin, 6.20*BL*/s^2^ with the 60^o^ open caudal fin, and 5.71 *BL*/s^2^ with the full-open caudal fin (Fig. 5(b)). As was also observed at 10 Hz, acceleration performance increased as the opening angle of the caudal fin decreased. The *u*_a_ difference between closed and full-open caudal fin is 18% (Fig. 5(b)). To reach the experimentally measured *u*_e_ (2.68 *BL*/s), 11.7 cycles were required with the caudal fin closed and 13.8 cycles with the full-open caudal fin (Fig. 5(b)). The difference between the full-open and closed caudal fins was 2.1 cycles, which was 0.0714 s, and the difference in swimming distance during this period was 0.16 *BL*. Furthermore, a difference of 0.16 *BL*/s was observed between the termination velocity *u*_a_ of the closed and full-open caudal fin (Fig. 5(b)). Figure 5 (c) and (d) compare the cost of transport and Froude efficiency between open and closed caudal fins. At both 10 Hz and 29 Hz, Froude efficiency improved as the opening angle of the caudal fin increased. Similarly, transport costs decreased with increasing caudal fin opening angle at both frequencies. At 10 Hz, closing the caudal fin increased transport costs by 8.2% and decreased Froude efficiency by 8.9% compared with the full-open caudal fin. In contrast, at 29 Hz, closing the caudal fin resulted in a 6.3% increase in transport costs and a 3.8% decrease in Froude efficiency relative to the full-open condition.

### Wake structure

Flow visualization revealed that at 10 Hz and 29 Hz, vortices generated from the dorsal fin reached the caudal fin, and their interaction contributed to vortex amplification. When the caudal fin was open, the reverse Karman vortex from the dorsal fin collided with the Karman vortex from the caudal fin, resulting in an enhanced vortex strength. Details are described below.

**Fig. 6:**
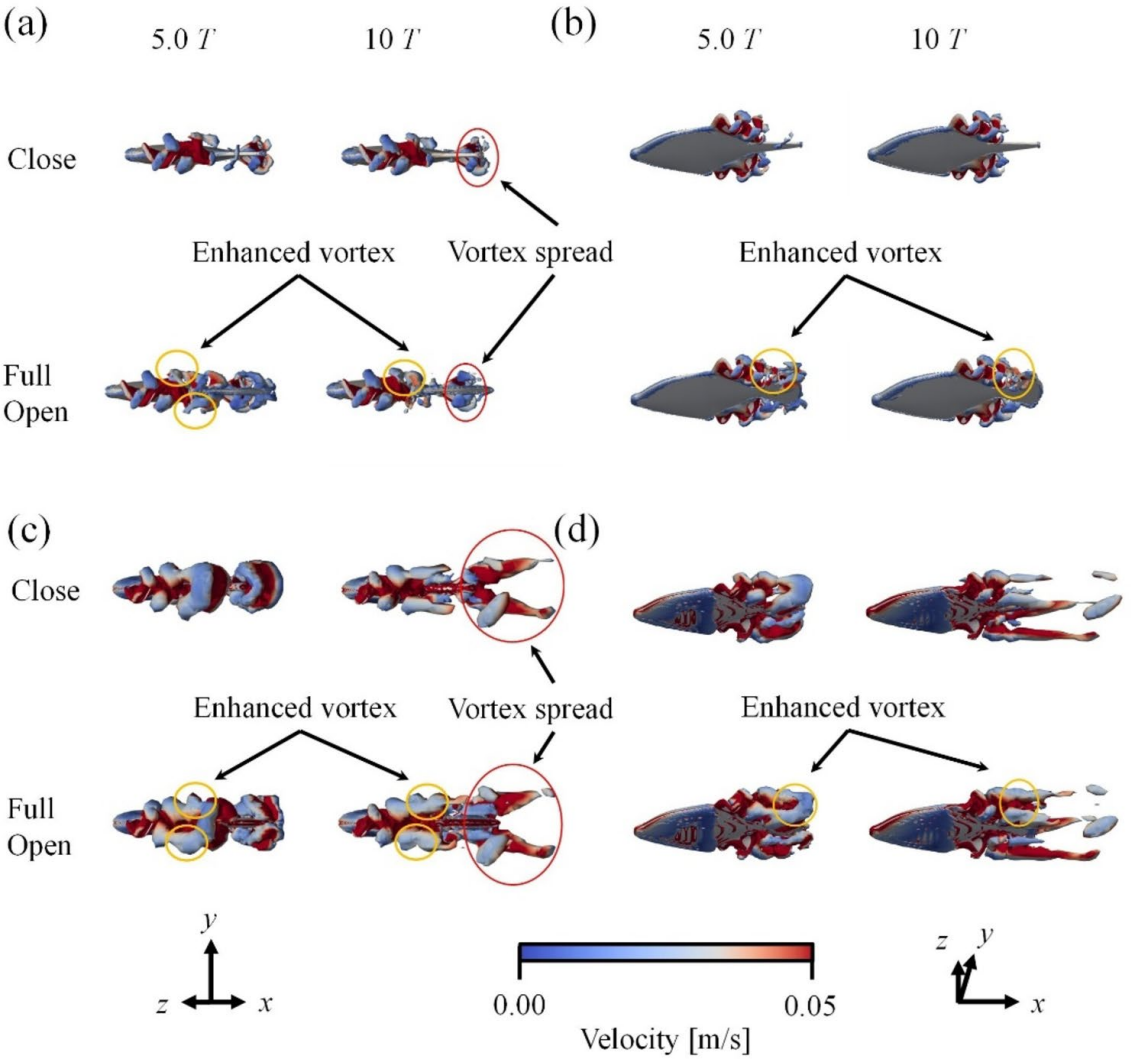
Three-dimensional wake structure of *R. ercodes* on (**a**) and, (**b**) *f* = 10 Hz, (**c**) and (**d**) *f* = 29 Hz. Differences in three-dimensional wake structure generated by *R. ercodes* owing to the opening and closing of the caudal fin.

**Fig. 7:**
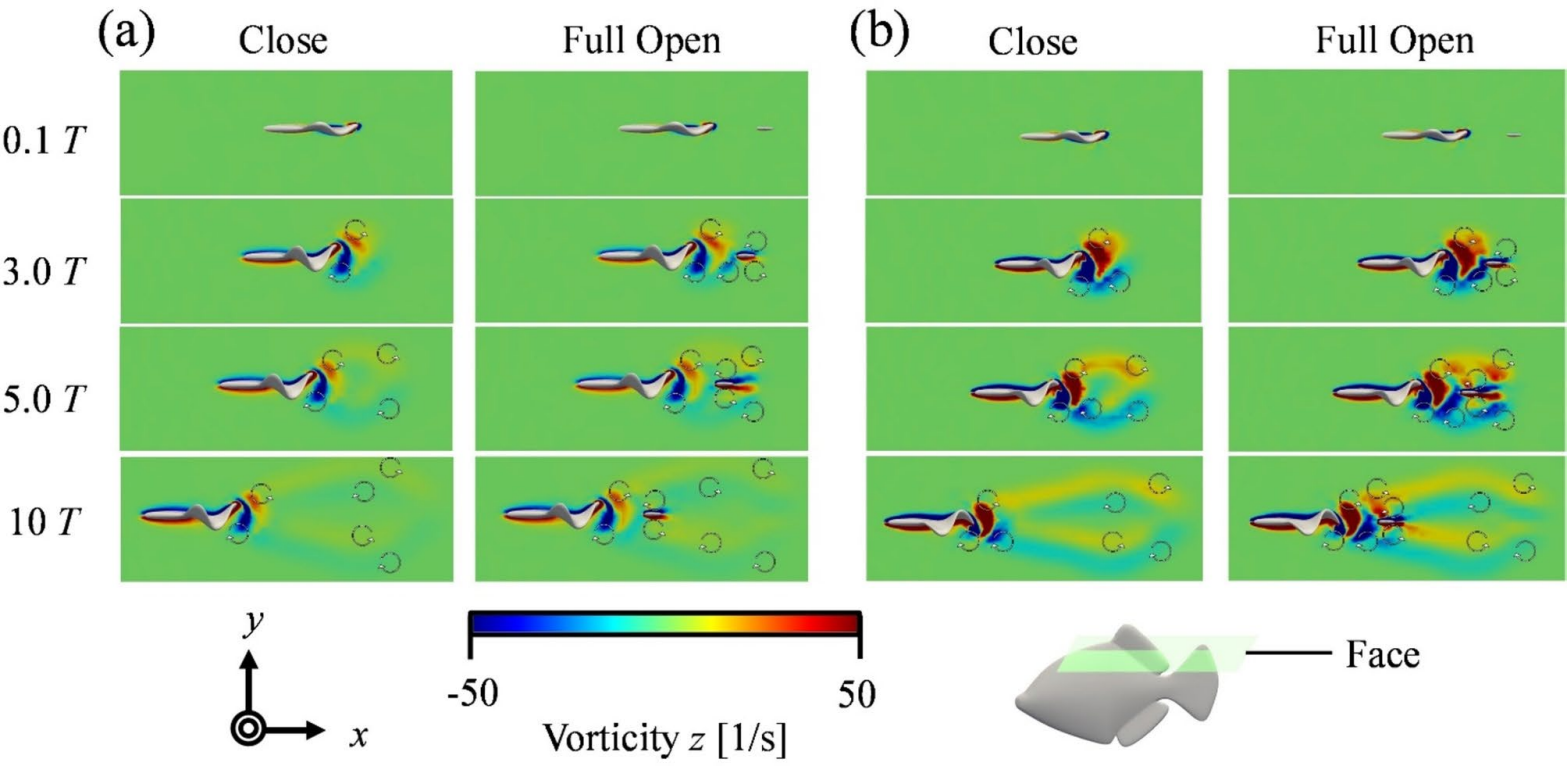
Compare the vorticity field from the dorsal fin surface (*z* = 0.01) by the opening and closing of the caudal fin. *f* = 10 Hz in (**a**) and *f* = 29 Hz in (**b**).

Figure 6 compares the flow field around *R. ercodes* when the caudal fin was full-open and closed. Here, we compare the flow fields between the full-open and closed caudal fin conditions, which showed the most significant differences. The flow fields for the caudal fin opening angles of 30° and 60° are presented in Supplementary Figures S7 to S10. Figure 6 (a) and (b) show the flow field when the frequency of the dorsal and anal fins was 10 Hz, and Fig. 6 (c) and (d) show it at 29 Hz. The flow field was visualized using the *Q*-criterion^50^. In Fig. 6, the value of the *Q*-criterion was visualized at 80. From Fig. 6 (b) and (d), it can be seen that the higher the frequency of the dorsal and anal fins, the larger the generated vortex. During the development of the vortex, the vortex originating from the dorsal fin does not reach the caudal fin within the first three cycles but reaches the caudal fin by the fifth cycle. This trend is seen at both 10 Hz and 29 Hz, regardless of the frequency of the dorsal and anal fins (Fig. 6 (a), (b), (c), (d)). Additionally, at the beginning of swimming with the caudal fin closed, a vortex is generated directly behind *R. ercodes*, and after acceleration, the vortex spreads over a wide area (Fig. 6 (a), (c)). Because the vortex is emitted in an oblique direction relative to the main flow, jet flows are generated at an oblique angle to the flow direction. The flow direction component of the jet flow becomes the thrust. This process is similar to the vortex development originating from the body and caudal fin of BCF swimming fish^43^ or the undulating fin^51^. On the other hand, when the caudal fin was full-open, the vortex generated directly behind *R. ercodes* at the start of swimming spread over a wide area during acceleration, and in doing so, it collided with the vortex generated from the caudal fin, forming a larger vortex (Fig. 6 (a), (b), (c), (d)). These results were similar for the anal and caudal fins (supplementary Fig. S4). Figure 7 shows cross-sectional views (*z* = 0.01) of the vorticity field around *R. ercodes* with the caudal fin full-open and closed. The blue vortices are clockwise, and the red vortices are counterclockwise. This indicates that a reverse Karman vortex originates from the dorsal fin, while a Karman vortex is generated from the caudal fin. Furthermore, by the third cycle, the reverse Karman vortex generated from the dorsal fin reaches the caudal fin. Then, it flows along the outside of the caudal fin, eventually colliding with the Karman vortex generated from the caudal fin (Fig. 6 3.0T-10T). The colliding reverse Karman vortex and Karman vortex interfered with each other in a strengthening manner, causing the reverse Karman vortex to expand, as shown in Fig. 6. These results were similar for the anal and caudal fins in the caudal fin (supplementary Fig. S5).

### Drag, thrust, and net thrust

**Fig. 8:**
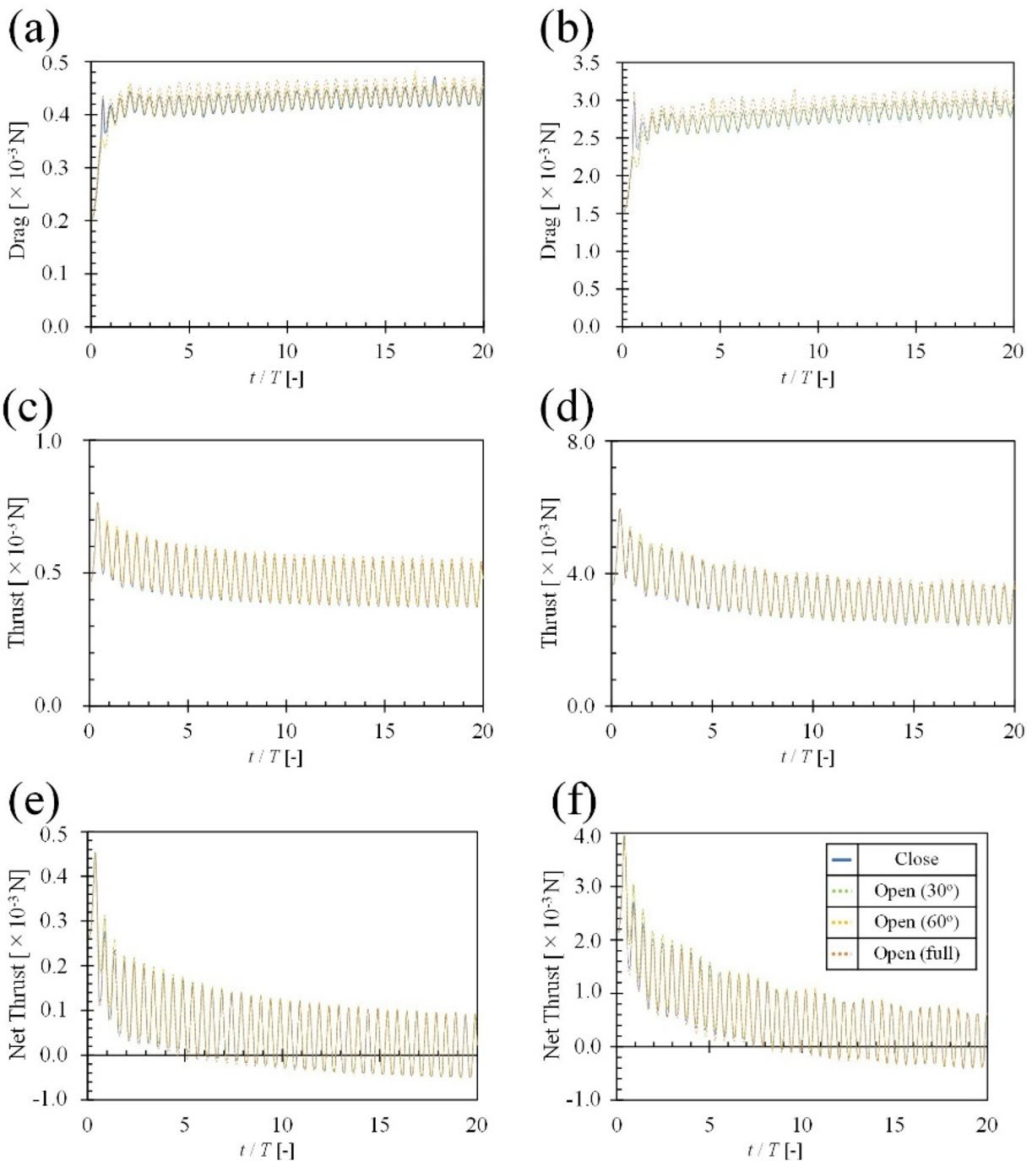
(**a**) and (**b**) show comparisons of drag between open and closed caudal fins. (**c**) and (**d**) show comparisons of thrust between open and closed caudal fins. (**e**) and (**f**) show comparisons of net thrust between open and closed caudal fins. In (**a**), (**c**), and (**e**), *f* of the dorsal and anal fins is 10 Hz. In (**b**), (**d**), and (**f**), *f* of the dorsal and anal fins is 29 Hz. The blue line shows the case where the caudal is closed, the green line shows the case where it is 30° open, the yellow line shows the case where it is 60° open, and the orange line shows the case where it is fully open.

Figure 8(a) compares the drag acting on *R. ercodes* at a frequency of 10 Hz, under four caudal fin conditions: closed, 30° open, 60° open, and full-open. Drag tended to increase with increasing caudal fin opening angle. From cycle 0.1 to cycle 20, closing the caudal fin decreased the average drag by 4.1%. Figure 8 (b) compares the drag acting on *R. ercodes* at a frequency of 29 Hz, under four caudal fin conditions: closed, 30° open, 60° open, and full-open. From cycle 0.1 to cycle 20, closing the caudal fin decreased the average drag by 4.7%. Figure 8(c) compares the thrust acting on *R. ercodes* at a frequency of 10 Hz, under four caudal fin conditions: closed, 30° open, 60° open, and full-open. From cycle 0.1 to cycle 4.9, closing the caudal fin decreased the average thrust by 1.7%, while from cycle 5.0 onwards, it decreased the average thrust by 3.4%. Figure 8(d) compares the thrust acting on *R. ercodes* at a frequency of 29 Hz, under four caudal fin conditions: closed, 30° open, 60° open, and full-open. From cycle 0.1 to cycle 4.9, closing the caudal fin decreased the average thrust by 0.9%, and from cycle 5.0 onwards, it decreased the average thrust by 3.6%. Figure 8(e) compares the net thrust acting on *R. ercodes* at a frequency of 10 Hz, under four caudal fin conditions: closed, 30° open, 60° open, and full-open. Closing the caudal fin increased the average net thrust by 6.6%. Figure 8(f) compares the net thrust acting on *R. ercodes* at a frequency of 29 Hz, under four caudal fin conditions: closed, 30° open, 60° open, and full-open. Closing the caudal fin increased the average net thrust by 7.3%.

## Discussion

We hypothesized that *R. ercodes* improve their average acceleration by closing their caudal fins. To verify our hypothesis, we examined two questions: (1) How did the opening and closing of the caudal fin affect the acceleration capability? and (2) what the mechanism is. Our results showed that the opening and closing of the caudal fins increased the average acceleration, supporting our assumption. Below are the answers to the two questions.

1. The results showed that closing the caudal fin improved the average acceleration regardless of *f*, increased the terminal velocity, and lowered the average drag (Fig. 5). On the other hand, when the caudal fin was full-opened, the average acceleration decreased, but the average thrust increased (Fig. 5).
2. Closing the caudal fin reduced the average drag due to the decrease in frictional drag from reduced surface area and the suppression of Karman vortices from the caudal fin, reducing pressure drag. Therefore, since the reduction in average drag from closing the caudal fin was more significant than the reduction in average thrust, the average net thrust increased, improving the acceleration index (Fig. 8). On the other hand, when the caudal fin was full-open, the reverse Karman vortices generated by the dorsal and anal fins are thought to have collided constructively with the Karman vortices from the caudal fin, strengthening the reverse Karman vortex and increasing the average thrust (Figs. 6 and 7).

### Effects of caudal fin opening and closing on R. ercodes hydrodynamic performance

We revealed the hydrodynamic performance of *R. ercodes* through a combination of water tank observation and 3D self-propulsion analysis of *R. ercodes* with dorsal and anal fin movements. 3D-CFD analysis showed that closing the caudal fin improved the average acceleration and the termination velocity *u*_*a*_ (Fig. 5). In addition, the average acceleration when closing the caudal fin was improved when *f* was 10 Hz compared to 29 Hz (Fig. 5). The termination velocity when closing the caudal fin was improved when *f* was 29 Hz than when *f* was 10 Hz (Fig. 5 (a) and (b)). Therefore, it is possible that caudal fin closing increases the average acceleration and termination velocity for feeding and migration at low velocities and enhances average acceleration plus more termination velocity to avoid danger when swimming at high velocities. Furthermore, the cost of transport required for swimming was increased when the caudal fin was closed, thus increasing the burden on *R. ercodes* (Fig. 5 (c) and (d)). To improve the average acceleration, instead of increasing the body part that moves, the caudal fin closes to reduce the area where drag acts. On the other hand, BCF swimming fish showed a negative correlation between swimming velocity and cost of transport^52^, which is similar to the present results. There were mechanistic differences between *R. ercodes* and BCF swimming fish; both increase net thrust when accelerating, but *R. ercodes* (MPF) reduces both thrust and drag, with drag decreasing more significantly, whereas BCF swimming fish increase both thrust and drag, with thrust increasing more significantly^53^.

### Mechanism of increased nut thrust through caudal fin closing

First, we consider the drag. As shown in Fig. 8 (a), when *f* was 10 Hz, closing the caudal fin of *R. ercodes* decreases the average drag by 4.1% from cycle 0.1 to cycle 20 compared with the full-open caudal fin. This is likely due to the larger surface area when the caudal fin is closed (2.47 × 10^− 3^ m^2^) compared to when it is full-open (2.18 × 10^− 3^ m^2^), resulting in increased frictional drag. Additionally, as shown in Fig. 7, Karman vortices are generated from the caudal fin when open, increasing pressure drag (supplementary Fig. S6). It is known that for fish, the formation of Karman vortices increases drag^54^. Consequently, closing the caudal fin of *R. ercodes* reduces drag. A similar trend is observed when *f* was 29 Hz (Fig. 7).

Next, we consider the thrust. As shown in Fig. 8 (c), when the oscillation frequency of *f* was 10 Hz, the average thrust of *R. ercodes* with the caudal fin closed was 1.7% lower from 0.1 cycles to 4.9 cycles compared with the full-open caudal fin. After 5.0 cycles, it becomes 3.4% lower. This shows no difference in the thrust in the early stages of swimming, but over time, the increase in thrust when the caudal fin is full-open is more significant than when the caudal fin is closed. As shown in Fig. 7, Karman vortices begin forming from the caudal fin after the third cycle when the caudal fin is full-open, reducing the thrust (Fig. 8 (c) and (d)). However, when the vortex generated from the dorsal fin reaches the caudal fin in the fifth cycle, the Karman vortex and the reverse Karman vortex from the dorsal fin collide to reinforce each other. This observation aligns with the results of Han, P. et al.^4^., which studied the interaction between the dorsal and caudal fins. Furthermore, the reverse Karman vortex generated from the dorsal fin, after colliding near the caudal fin, expands around the caudal fin and flows farther back (Fig. 6). As a result, with the caudal fin full-open, the average thrust increased by 5.7% compared to when the caudal fin was closed (Fig. 8 (c)). A similar trend was observed when *f* was 29 Hz (Fig. 6).

Finally, based on the above results, we examine the net thrust. When *f* was 10 Hz, closing the caudal fin in *R. ercodes* decreased the average thrust after the fifth cycle and reduced the average drag. Since the decrease in average drag was more significant than the decrease in average thrust, the average net thrust increased by 6.6%. When *f* was 29 Hz, the average net thrust increased by 7.3% through a similar mechanism.

### Limitations and future works

The elasticity of the undulating fins (dorsal and anal fins) was not considered in this study. However, we used equations showing displacement extracted from actual undulating fin movements reported in previous studies. Although the values of velocity and so on may change when softness is considered because the same motion is used in the model with the caudal fin open and the model with the caudal fin closed, it is expected that the relative relationship in which the resistance is small when the caudal fin is closed will not change, and this issue does not affect the main claim that closing the caudal fin can accelerate faster. The caudal fin is generally used to change direction^23^, but we did not consider it in this study. In addition, the present study focused on acceleration movements and did not cover cruising swimming. However, as the *R. ercodes* we observed in captivity exhibited open caudal fins during cruising swimming, it is possible that they open their caudal fins to enhance Froude efficiency during cruising. Therefore, it is necessary to investigate the effects of caudal fin opening and closing on postural control and during cruising swimming to clarify why *R. ercodes* open and close their caudal fins.

### Biological implications and application to underwater robots

Studies on fin opening and closing have been conducted for many years^19,22–24^. However, as far as we know, no studies have examined the effects of caudal fin opening and closing on linear swimming in MPF swimming fish. We performed 3D-CFD analysis on *R. ercodes* performing balistiform, a type of MPF locomotion, and found that closing the caudal fin improved the average acceleration, increased transport cost, and decreased the dimensionless Froude efficiency. These findings will contribute to understanding the ecology and behavior of fish with caudal fins that can open and close, as the ability to accelerate is crucial for fish survival^25^.

The results of this study may also be helpful to improve the acceleration capability of underwater robots. Several underwater robots have been developed to enhance locomotion performance using fin interactions. For example, adjusting the phase difference between the anal fin and the caudal fin can suppress head oscillation, while deploying both the dorsal and anal fins can increase thrust. Moreover, MPF-type underwater robots have also been developed, and our findings suggest that incorporating a caudal fin deployment mechanism into robots using MPF locomotion could enhance their acceleration performance^55^.

## Conclusion

Tank observations and fluid dynamic analysis revealed that closing the caudal fin can reduce the cost of transport and improve acceleration performance in *R. ercodes.* The key findings obtained are summarized below.

1. Regardless of frequency, acceleration performance increased as the opening angle of the caudal fin decreased (Fig. 5 (a), (b)).
2. Propulsive efficiency increased, and the cost of transport decreased with increasing caudal fin opening angle, irrespective of frequency (Fig. 5 (c), (d)).
3. When the caudal fin is opened, the reverse Karman vortices shed from the anal and dorsal fins are reinforced by the Karman vortices generated from the caudal fin (Figs. 6 and 7). As a result of this reinforcement, propulsive efficiency was enhanced, and the cost of transport was reduced (Fig. 5).
4. While thrust decreases as the caudal fin is closed, the accompanying reduction in drag is more significant, leading to enhanced acceleration performance with decreasing fin opening angle (Fig. 8).

## Electronic supplementary material

Below is the link to the electronic supplementary material.

Supplementary Material 1

Supplementary Material 2

Supplementary Material 3

## Data Availability

We conducted CFD analysis using OpenFOAM; it is open-source, so anyone can get the numerical analysis code. Moreover, the datasets and codes used for the analysis is available from the corresponding author upon request.
