# Supplementary material for: Relationship between caudal fin closing motion and acceleration capability of Rudarius ercodes balistiform locomotion
Source: Sci Rep. 2025 May 8;15:16045. doi: 10.1038/s41598-025-00315-9 (PMC12062403; doi:10.1038/s41598-025-00315-9)
Supplement: Supplementary file 3 — Supplementary Material 3 [file 41598_2025_315_MOESM3_ESM.docx]

The Electronic Supplementary Materials (ESM) in the article " Relationship between Closing Motion of Caudal Fin and Acceleration Capability of Balistiform Locomotion of Rudarius ercodes"

The file contains:

1) The model and the methods of verification and validation,

2) ESM references.

3)Legends for supplementary videos

4) Figures S1, S2, S3, S4, S5, S6,

**The analytical target and methods of CFD analysis**

To evaluate the validity of this analysis, we performed a simulation analysis of a typical self-propulsion problem, anguilliform, and compared it with previous studies by Kern et al.^[s1]^ and Huang et al.^[s2]^. The model, motion, and Reynolds number used in this analysis are the same as those used in previous studies and are discussed in detail below. CFD simulations were performed using the open source finite volume CFD toolbox OpenFOAM^®^-v1806^[s3]^ to calculate the drag coefficient, swimming velocity in the *x*-direction, and swimming velocity in the *y*-direction and compared with previous studies. The main manuscript stated that the governing equations were the continuity and three-dimensional incompressible Reynolds-averaged Navier-Stokes equations. The Reynolds number was defined as;

$R_{e}=\frac{\rho U_{\mathbb{I}}}{\mu}$ , (S1)

Where kinematic viscosity is μ = 1.4×10^-4^ m^2^/s, density is ρ = 1000 kg/m^3^, and *U*_Ⅱ_ is the resulting velocity. The Reynolds number averages 2562, similar to Kern et al.'s^[s1]^ result. Considering this, we used laminar models^[s4]^.

Overset grid method was used in this study. The overset grid method was used in this study. The overset is a generic implementation of overset meshes for static and dynamic cases. This method allows for complex mesh motions without the penalties associated with mesh deformation^[s5]^. The calculation volume was 10*L* in length, 2.4*L* in height, and 2.4*L* in width (Fig. S1). A hexahedral volume mesh was created using the snappyHexMesh of OpenFOAM. The fluid region was made with fine meshes around the analysis target and coarse meshes in the outlying areas; a 1-layer boundary layer mesh was created around the analysis target. A constant flow speed of 0 m/s was applied at the inlet boundary. The average static relative pressure was set to 0 Pa at the outlet boundary. As the boundary condition, non-slip wall boundaries were used for the wall of the surface of the 3D model, and free flow-out boundaries were used for the other walls. A program was created to calculate velocity from the forces acting on the surface of the 3D model and implemented as a library in OpenFOAM to account for the reaction and inertia forces from the fluid. This paper considered the x-axis- and *y-axis* displacements in Kern et al.^[s1]^ and Huang et al.^[s2]^. The time step during the simulation was adjusted according to the maximum courant number 0.2.

The 3D models used in the simulations were created using Blender 2.93.6.^[s6]^ according to the parameters reported by Kern et al.^[s1]^. The diagram of the model is shown in Fig. S2. The body's width, *d*, is divided into three pieces, as in equation (S2).

$d=\left\{ \begin{aligned} \sqrt{s\left( 2d_{h}-s \right)} 0\leq s<s_{h} \\ d_{h}-\left( \frac{s-s_{h}}{s_{t}-s_{h}} \right)^{2}\left( d_{h}-d_{t} \right) s_{h}\leq s<s_{t} \\ \frac{L-s}{L-s_{t}}d_{t} s_{t}\leq s\leq L \end{aligned} \right.$ （S2）

where *d*_h_=*s*_h_=0.04*L*, *s_t_*=0.95*L*, and *d*_t_=0.01*L* and *L* is the body length set as *L* = 1 m. Subscripts *h* and *t* represent the fish's head and tail, respectively.

The height *W*_(s)_ with elliptic curve shape is as in equation (S3).

$W_{\left( s \right)}=b\sqrt{1-\left( \frac{s-a}{a} \right)^{2}}$ (S3)

where the two half axes *a* and *b* are set as *a* = 0.51 *L* and *b* =0.08 *L*, respectively.

(S4) equation describes the lateral displacement of the mid-line in a local coordinate system.

$y_{s}\left( s,t \right)=A_{\max}L\frac{0.03125+\frac{s}{L}}{1.03125}sin\left[ 2\pi\left( \frac{s}{L}-\frac{t}{T} \right) \right]$ (S4)

To verify our results with the study of Kern et al.^[s1]^, the coefficient of motion amplitude *A*_max_ is set as *A*_max_ = 0.125 m, and the cycle time is *T* = 1 s.

In the results, the drag coefficients, and longitudinal velocity, are compared with the previous studies, as shown in Fig. S3. The longitudinal velocity calculated by this analysis method was 0.42 m/s, which had an error of 5 % from the two previous studies. The period of maximum drag coefficient is out of line with previous studies. However, the cycle average is 0.5 seconds, which is consistent with previous studies. Furthermore, as shown in Fig. S3 (c), the relationship between swimming speed and thrust coefficient is similar to previous studies. This is thought to be due to the different methods of controlling the first period. Since the difference in control during the first cycle is expected to be due to the difference in control during the first cycle, we do not consider this difference problematic for validity considerations. Therefore, the error in the longitudinal velocity is 5%, and the period of the drag coefficient is consistent, so the validity of this analysis method is sufficient.

**References**

1. Kern, S., & Koumoutsakos, P. Simulations of optimized anguilliform swimming. Journal of *Experimental Biology*. 209(24), 4841-4857 (2006).
2. Huang, X. T., Sun, P. N., Lyu, H. G., & Zhong, S. Y. Study of 3D self-propulsive fish swimming using the δ+-SPH model. *Acta Mechanica Sinica*. 39(1), 722053 (2023).
3. OpenCFD. OpenFOAM^®^ - Official home of The Open Source Computational Fluid Dynamics (CFD) Toolbox. <https://www.openfoam.com/>. (Accessed 7 February 2024).
4. OpenFOAM: user guide: laminarModel< BasicTurbulenceModel > Class Template Reference. Electronic version. <https://www.openfoam.com/documentation/guides/latest/api/classFoam_1_1laminarModel.html>. (Accessed 7 February 2024).
5. OpenFOAM: user guide: Overset. Electronic version. <https://www.openfoam.com/documentation/guides/latest/doc/guide-overset.html>. (Accessed 7 February 2024).
6. Blender Foundation. blender. org—Home of the Blender project—Free and Open 3D Creation Sofware. https://www.blender.org/. (Accessed 7 February 2024).

**Legends for supplementary videos**

Supplementary Video 1. *R. ercodes* accelerates from a stationary state by closing its caudal fin (0.2x speed).

Supplementary Video 2. Created motion of *R. ercodes* from the left side.

Figure S1. Analytical region and model.

Figure S2. (a) is a diagram showing the analytical shape of the 3D fish^s1^. Height *W*_(s)_ (bottom panel) and half-width *d* (top panel) in the longitudinal direction according to Eqs. (2) and (3), respectively. (b) is the 3D model used to solve the fluid dynamics part of the fluid–body interaction.

Figure S3. Analysis Results. (a) is the change in drag coefficient with time. (b) is the change in longitudinal velocity with time. (c) The right axis is the change in drag coefficient with time. The left is the change in longitudinal velocity with time.

Figure S4. Three-dimensional wake structure of *R. ercodes* on (a) and (b) *f* = 10 Hz, (c) and (d) *f* = 29 Hz. Differences in three-dimensional wake structure generated by *R. ercodes* owing to the full opening and closing of the caudal fin.

Figure S5. Compare the vorticity field from the anal fin surface (*z* = -0.009) by the opening and closing of the caudal fin. *f* = 10 Hz in (a) and *f* = 29 Hz in (b).

Figure S6. Compare the pressure from the dorsal fin surface (*z* = 0.01) by the opening and closing of the caudal fin. *f* = 10 Hz in (a) and *f* = 29 Hz in (b).

Figure S7. Three-dimensional wake structure of *R. ercodes* on (a) top view, (b) oblique top view. (Caudal fin is 30^o^)

Figure S8. Compare the vorticity field from the anal fin surface (*z* = 0.003). *f* = 10 Hz in (a) and *f* = 29 Hz. (Caudal fin is 30^o^)

Figure S9. Three-dimensional wake structure of *R. ercodes* on (a) top view, (b) oblique top view. (Caudal fin is 60°)

Figure S10. Compare the vorticity field from the anal fin surface (*z* = 0.005). *f* = 10 Hz in (a) and *f* = 29 Hz. (Caudal fin is 60°)
